# Development of New Method for Simultaneous Analysis of Piracetam and Levetiracetam in Pharmaceuticals and Biological Fluids: Application in Stability Studies

**DOI:** 10.1155/2014/758283

**Published:** 2014-07-08

**Authors:** Farhan Ahmed Siddiqui, Nawab Sher, Nighat Shafi, Alisha Wafa Sial, Mansoor Ahmad, Mehjebeen, Huma Naseem

**Affiliations:** ^1^Faculty of Pharmacy, Federal Urdu University Arts, Science and Technology, Karachi 75300, Pakistan; ^2^Faculty of Pharmacy, Ziauddin University, Karachi 75600, Pakistan; ^3^Department of Chemistry, University of Karachi, Karachi 75270, Pakistan; ^4^Department of Environmental Sciences, Federal Urdu University Arts, Science and Technology, Karachi 75300, Pakistan; ^5^Faculty of Medicine, Ziauddin University, Karachi, Pakistan; ^6^Department of Pharmacognosy, Research Institute of Pharmaceutical Sciences, Faculty of Pharmacy, University of Karachi, Karachi 75270, Pakistan

## Abstract

RP-HPLC ultraviolet detection simultaneous quantification of piracetam and levetiracetam has been developed and validated. The chromatography was obtained on a Nucleosil C18 column of 25 cm × 0.46 cm, 10 *μ*m, dimension. The mobile phase was a (70 : 30 v/v) mixture of 0.1 g/L of triethylamine and acetonitrile. Smooth flow of mobile phase at 1 mL/min was set and 205 nm wavelength was selected. Results were evaluated through statistical parameters which qualify the method reproducibility and selectivity for the quantification of piracetam, levetiracetam, and their impurities hence proving stability-indicating properties. The proposed method is significantly important, permitting the separation of the main constituent piracetam from levetiracetam. Linear behavior was observed between 20 ng/mL and 10000 ng/mL for both drugs. The proposed method was checked in bulk drugs, dosage formulations, physiological condition, and clinical investigations and excellent outcome was witnessed.

## 1. Introduction

**Figure 1:**
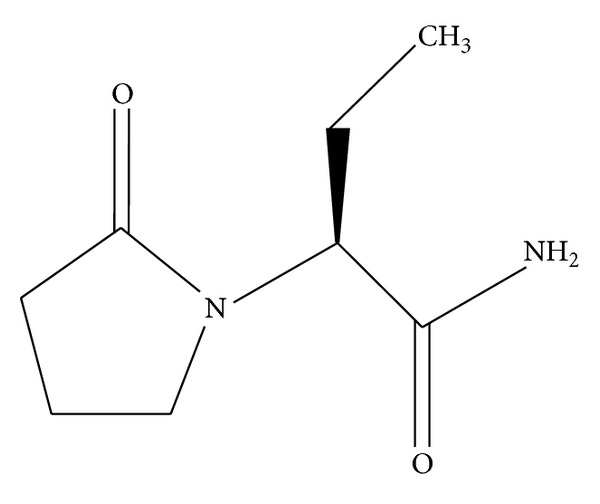
Structure of levetiracetam.

Levetiracetam chemical name is (*S*)-ethyl-2-oxo-1-pyrrolidineacetamide (Figure 1). It is a novel antiepileptic drug which is quite unique as compared to other antiepileptic drugs, being structurally analogous to piracetam, although exact mechanism of its action is yet to be established. However, it is believed that it binds to a synaptic vesicle protein, thus slowing down nerve conduction across synapses [1].

**Figure 2:**
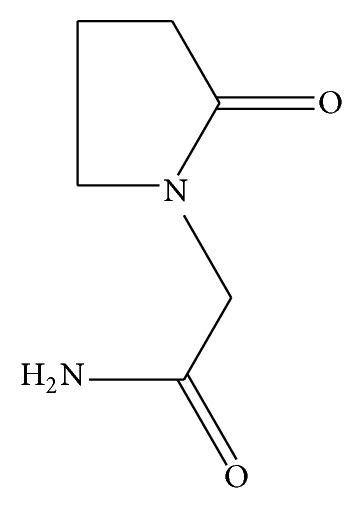
Structure of piracetam.

Piracetam (Figure 2) is chemically designed as 2-oxo-1-pyrrolidine acetamide. It is a nootropic, psychopharmacological drug. It is believed that it has a role in augmenting cognition and memory, impeding brain disaster, and improving blood and oxygen flow to the brain. It has been known to enhance mental conditions like dementia, dyslexia, and Alzheimer's disease and Down syndrome [2].

Stability studies are regulatory requirement. It provides proof of the safety and integrity of the product throughout its shelf life. Variety of environmental factors such as humidity, temperature, and light can have a detrimental role on quality of the product. Therefore, understanding of stability of a product helps to understand the long term impact of environment on the drug product. Stability studies suggest proper formulations, design manufacturing processes, and selecting proper storage condition and packaging for the drug product. Furthermore, it helps in establishing shelf life of product [3–5]. All these make it highly important to have a stability indicating method in hand.

Literature assessment disclosed that levetiracetam has been estimated by various methods in pure and pharmaceutical dosage forms including HPLC-UV [6–11] and HPLC-MS [12, 13], while piracetam by HPLC, micellar electrokinetic chromatography, thin layer densitometry and capillary electrophoresis, and so forth [14–25].

But simultaneous determination of both drugs has not been reported yet. Only Hadad et al., [26] described a simultaneous assay method but it is based on chemical reaction subjected to various factors and sensitivity and specificity of the method cannot be achieved. HPLC-UV is the most commonly available sophisticated technique, utilized to develop a simultaneous quantification of piracetam and levetiracetam. The objective of the current study was to develop an accurate, simple, and economical analytical method for the estimation of piracetam and levetiracetam in pharmaceutical dosage forms as well as in biofluids. This endeavor concluded in development of a trouble-free, fast, perfect, and sensitive HPLC method with commonly available UV detector for the concurrent estimation of levetiracetam and piracetam in pure form and pharmaceutical formulations.

## 2. Experimental

### 2.1. Materials and Reagents

Piracetam and levetiracetam were kind gifts from National Pharmaceuticals, sulphuric acid was purchased from Merck (Germany), and methanol (HPLC grade) was from Fisher Scientific. The tablets containing piracetam and levetiracetam were purchased from home market. Purified and double distilled and deionized water was used throughout this study, produced by passing through RO plant (Waterman, Pakistan) and further filtered through a 0.45 *μ*m membrane filter (Millipore, Bedford, MA, USA).

### 2.2. Apparatus

A UV-visible Shimadzu 1650 PC spectrophotometer with UV Probe software, Sartorius TE2145 analytical balance, Jenway 3240 pH meter, Elmasoni E 60 H Ultrasonic cleaner, and class A amber glass flasks were used in this research. For chromatography a Shimadzu LC 20 AT pump, SIL 10A autoinjector, SPD 20A prominence UV/VIS detector, and SCL 10A system controller with LC Solutions software were used. Separation was executed on a Hibar *μ* Bondapak ODS C18 HPLC column (4.6 × 250 mm; 5 *μ*m bead size) uphold at 25°C.

### 2.3. Chromatographic Conditions

The HPLC analysis was carried out at ambient temperature. Mobile phase consisting of triethylamine (10 mL in 1000 mL of water) and acetonitrile (70 : 30 v/v), adjusted to pH 6.5 ± 0.2 by phosphoric acid. Prior to use mobile phase was filtered through a 0.45 *μ*m membrane filter (Millipore, Bedford, MA, USA). Flow rate was 1.0 mL/min Injection, injected volume was 20 *μ*L, and the detecting wavelength was 205 nm. Mobile phase was used as extraction solvent as well.

### 2.4. Analytical Procedure

#### 2.4.1. Standard Preparation

10 mg weight each of piracetam and levetiracetam was taken in a 100 ml volumetric flask separately. 50 mL of the diluent was added, sonicated for few minutes and stirred for 30 minutes on magnetic stirrer to dissolve the sample completely. The sample was diluted up to the mark level to produce 100 *μ*g/mL of each piracetam and levetiracetam. Before injection into HPLC system, sample was filtered through 0.45 *μ*m membrane filter paper.

#### 2.4.2. Assay of Tablets Containing the Drug

For making sample of 100 *μ*g/mL of piracetam and levetiracetam each, 20 tablets were weighed and grounded to get homogenized powder. The sample weight equivalent to 100 mg of piracetam and levetiracetam was accurately taken in 100 mL volumetric flask, followed by the addition of 50 mL of diluent. The sample after sonication for 10 minutes was placed for stirring for 20 minutes. After attaining room temperature the sample was diluted up to the mark level with same diluent. Solution was filtered and 10 mL of clear filtrate was further diluted to 100 mL to obtain final concentration.

Further dilutions were made according to the required concentration. All the samples prepared were filtered through 0.45 *μ*m membrane filter paper, before injecting into the HPLC system.

#### 2.4.3. Assay of Syrup Containing Drugs

Syrup containing 10 mg of piracetam and 10 mg of levetiracetam were taken in 100 ml volumetric flask, 50 mL of dilution solvent was added and sonicated for few minutes, sample was stirred on magnetic stirrer for 15 minutes and diluted up to the mark level with same diluent. Sample was filtered through 0.45 *µ*m filter paper to obtain clear solution. Further dilutions were made to produce the required concentration.

#### 2.4.4. Urine Drug Analysis

Urine samples, from twenty-five healthy volunteers were collected and were stored at 4°C for 24 hrs. 10 mL of stock solution was taken in 50 mL volumetric flask and 15 mL of diluent was added followed by 25 mL of urine sample. The sample after sonication for 05 minutes was stirred for 10 minutes to cool down the temperature and then diluted up to the mark level. Further dilutions were made. The sample after passing through 0.45 *μ*m membrane filter paper was injected into the HPLC system.

#### 2.4.5. Serum Drug Analysis

For analysis in plasma samples blood samples were procured from healthy volunteers and concentrations of piracetam and levetiracetam in plasma were analyzed by the proposed method. 2 mL of plasma was mixed with 2 mL of acetonitrile and 2 mL of drug solution was shaken, vortexed for 5 min, and centrifuged at 3000 rpm for 10 min. supernatant clear liquid was mixed from both the drugs and was appropriately diluted to get working range concentrations of piracetam and levetiracetam. After filtration through 0.45 mm membrane filter paper the samples were injected into the HPLC system for quantization.

#### 2.4.6. Pharmacokinetics Studies

This study was carried out in male healthy Pakistani individuals, bearing age range from 18 to 28 years and body weight of volunteers was from 55 to 75 kg. All the involving volunteers were educated about the aim of the study and all the risk associated. A written and signed informed consent statement was taken from all the volunteers. Local ethical committee approved the study protocol. Volunteers were medically examined, and laboratory examination (blood biochemistry, haematology, and urine) was completed before the study to ensure the suitability of the experiment; the volunteers were forbidden from taking any medicine for 2 weeks proceeding to and throughout the study period. After an overnight fasting, piracetam 800 mg tablet and levetiracetam 500 mg as a single dose were given to subject individually with 250 mL of water. Only water was allowed to be taken until 2 hours of intake. After which scheduled breakfast, lunch, and dinner were provided to all volunteers. Volunteers were prohibited from any active work. Through an indwelling cannula about 3 mL blood samples were drawn into heparinized tubes according to a scheduled time scale. That is, before (0 h) and at 0.5, 1, 1.5, 2, 2.5, 3, 3.5, 4, 4.5, 5, 5.5, 6, 6.5, 7, 7.5, 8, 9, 10, 15, 20, 25, 30, and 35 hr after dosing of levetiracetam, while blood was taken at 0.5, 1, 1.5, 2, 2.5, 3, 3.5, 4, 4.5, 5, 7, 9, 12, 15, 20, 25, and 30 hr after dosing of piracetam. And Plasma was separated out by centrifuging the samples at 5000 rpm for 10 min and stored frozen at −20°C in sterilized glass vials.

## 3. Result and Discussion

Analytical method development for drugs substances and its validation have very recently received significant importance owing to their substantial importance in quality control laboratories of pharmaceutical and chemical Industries and drug development. Generally an HPLC analytical method is associated with complex and sophisticated equipment, personal skills in chromatographic techniques, and qualification for use and disposal of solvents. This study was aimed at development of a straightforward, fast, and at the same time a highly sensitive, accurate, and precise HPLC method for the simultaneous determination of piracetam and levetiracetam in bulk, formulations, and human serum accommodating the most frequently employed C-18 column and UV detection. Literature survey did not come up with a single report describing quantification of these drugs in simultaneous mood, which prompted the development of an analytical method which could analyze these drugs simultaneously.

### 3.1. Method Optimization

Various experiments were performed to develop a proper mobile phase for effective separation of piracetam and levetiracetam. Mobile phase composition, stationary phase, flow rate, and detecting wavelength and pH of mobile phase have been excessively worked out to optimize the developed method. Early experiments were done with methanol and water mixtures with regard to mobile phases and broad peaks were recorded; after many trials methanol was replaced with acetonitrile in different ratio with water as mobile phase.

Different ratios of water and acetonitrile (10 : 90, 20 : 80, 30 : 70, 40 : 60, 50 : 50, 60 : 40, 70 : 30, 80 : 20, and 90 : 10) were tried. An unacceptable tailing factor for peaks of both drugs was observed. In RP-HPLC peak tailing is common due to interaction between positively charged amine and an acid silanol of stationary phase surface; normally acidic and neutral analytes do not interact as compared to basic compounds. It was thought after several trials with acetonitrile and water as mobile phase that positively charged amine in both analytes may interact with silanol of stationary phase and it may cause of peak tailing. After several trials, the peak tailing for both the drugs triggered us to add triethylamine into mobile phase. Triethylamine strong interaction with silanol inhibited it to interact with nitrogen group of amide function in our drugs, which helped in reduction of peak tailing factor. Different concentrations of triethylamine were used and its addition up to 0.1 g/L steadily improved the chromatographic behavior. Optimum condition was reached after several trials and acetonitrile and 0.1 g/L aqueous solution of triethylamine adjusted to pH 6.7 in the proportion 30 : 70 (v/v) were found to be a suitable mixture for separation of piracetam and levetiracetam.

### 3.2. Method Validation

Method validation institutes the performance characteristics which confers suitability of the method for its intended use. ICH [27] guideline was followed to perform validation studies and various parameters like system suitability, specificity, linearity, accuracy, precision, and sensitivity were tested by experimenting on the samples matrix.

System suitability is an integral part of method development program and is performed prior to every analysis to ensure the suitability of the system on which validation is to be carried out. For system suitability five replicates of drug samples were run at 100 ng·mL^−1^ concentration level. Repeatability with respect to peak height and peak area in five replicates, peaks symmetry (tailing factor), resolution between the peaks of drugs, theoretical plates of the column (column efficiency), capacity factor, and relative retention time were calculated. Specificity is the characteristic feature of the method by which it isolates and quantifies the analyte in presence of other components that might be present. Levetiracetam and piracetam were spiked in excipients, used in formulation products, and tested for quantification which demonstrated excellent specificity. Seven different concentrations ranging from 20 to 10000 ng mL^−1^ for both analytes were evaluated for establishment of linearity. Recovery studies were performed on formulations samples at three different concentration levels (80%, 100% and 120%); each sample was made in triplicate. Similarly the study was extended to spiking of drugs into excipients, human urine, and blood serum and accuracy of the method was presented as the average recovery obtained. Multiple testing was done on the same homogeneous bulk sample to evaluate the repeatability on two nonconsecutive days to demonstrate within day and day to day variations. LOD and LOQ were evaluated by the empirical formulas mentioned in ICH guidelines. Assay study was carried out in two different labs with two different instruments in order to establish robustness of the method. Lab 1 was Faculty of Pharmacy, Federal Urdu University of Arts Science and Technology, while lab 2 was in the Research Institute of Pharmaceutical Sciences, Faculty of Pharmacy, University of Karachi.

#### 3.2.1. Selectivity and Specificity

**Figure 3:**
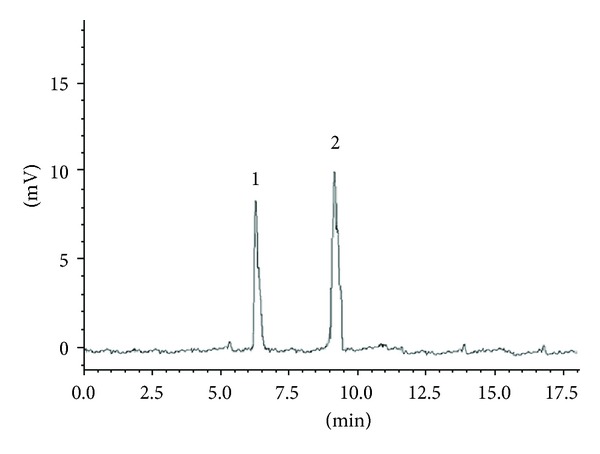
Typical chromatogram of standard showing separation of piracetam 100 ng/mL (1) and levetiracetam 100 ng/mL (2).

**Figure 4:**
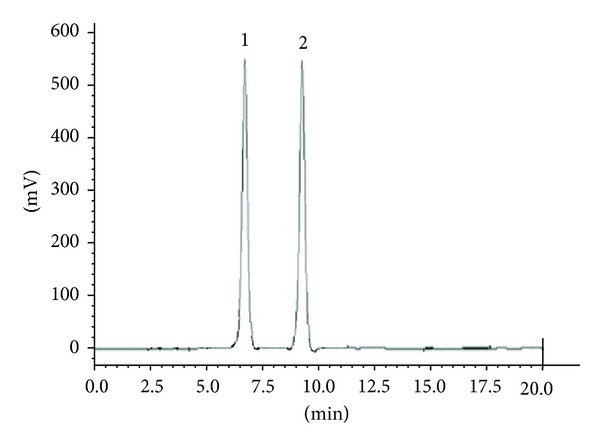
Typical chromatogram of pharmaceutical formulation showing separation of piracetam 5000 ng/mL (1) and levetiracetam 5000 ng/mL (2).

Piracetam and levetiracetam separation was measured in term of resolution factor of the peaks which in turn establish the selectivity of the method. Quantification of these drugs in presence of other components demonstrated excellent specificity and proposed method produced selective isolation and no interference from excipients was noticed. No other endogenous peaks were observed (Figures 3-4).

We also studied interference of some other antiepileptic drugs like phenytoin, phenobarbital, and carbamazepine, but all the mentioned drugs were not eluted in given chromatographic condition up to 10 minutes, so it can be concluded that newly developed method is specific.

#### 3.2.2. Linearity

Linearity of the method was studied in concentration range of 20–100000 ng/mL of piracetam and levetiracetam and calibration curve generated gave a linear response with correlation coefficient (*r*2) of >0.9990 for both analytes.  Table 1 shows the linear regression equations of piracetam and levetiracetam individually, using six replicate analyses and Table 2 shows the results of linear regression equation.

#### 3.2.3. Accuracy and Precision

Precision and accuracy were studied in the concentration range 800–1200 ng/mL for piracetam and levetiracetam. Results are portrayed in Table 3. Accuracy and precision varied between 99.47 and 101.75% and between 0.97 and 1.45%, respectively. Within day and day to day precision found were from 1.07 to 2.33% and from 0.77 to 1.57, respectively (Table 4). All the values of accuracy and precision are within acceptable domain.

#### 3.2.4. Ruggedness

Study carried out in different laboratories indicated ruggedness of the method and there was no notable deviation of results from the acceptable limits.

#### 3.2.5. Limit of Detection and Quantification

Limit of detection is the lowest limit of analyte to be detected not necessarily quantitated in a sample, while quantification limit is the lowest amount of analyte which can be quantitated with considerable degree of accuracy and precision. The LOD and LOQ are evaluated in Table 2.

#### 3.2.6. Recovery from Plasma

**Figure 5:**
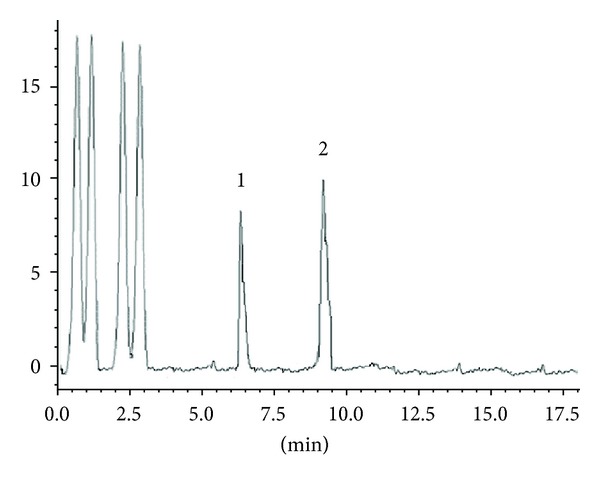
Typical chromatogram of drugs spiked with human serum showing separation of piracetam 100 ng/mL (1) and levetiracetam 100 ng/mL (2).

**Figure 6:**
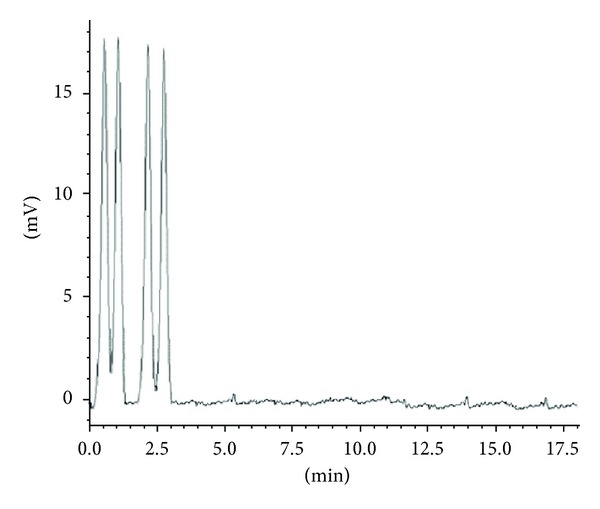
Typical chromatogram of blank serum.

Plasma samples were spiked with both of these drugs at mentioned concentration level so as to understand the applicability of the proposed method in human serum. Six different concentration level samples over the established range, each in triplicate, were assessed. Percent recovery obtained for both analytes is expressed in Table 5. Regardless of the drugs concentration, the percent recoveries obtained were in ranged of 96.8 to 99.84%. Typical chromatogram of drug spiked with human serum and blank serum is expressed in Figures 5 and 6, respectively.

#### 3.2.7. Recovery from Urine

**Figure 7:**
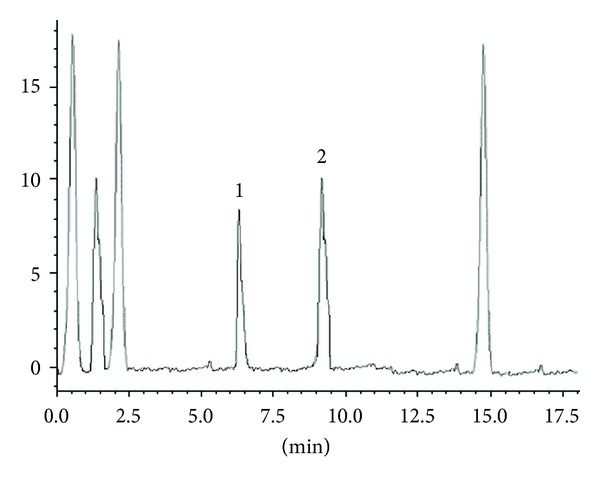
Typical chromatogram of drugs spiked with human urine showing separation of piracetam (1) and levetiracetam (2).

**Figure 8:**
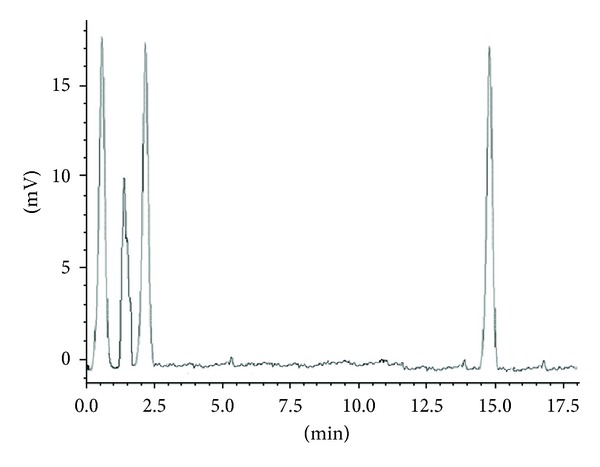
Typical chromatogram of blank urine.

Applicability of the proposed method in human urine was evaluated by spiking urine samples with both of these drugs in mentioned concentration level and assayed in triplicate. Method application in urine sample was tested according to the mentioned protocol above and the % recoveries produced are shown in Table 5. There was no considerable difference among the mean % recovery of both of these drugs in urine samples. Typical chromatogram of drug spiked with human urine and blank urine is shown in Figures 7 and 8, respectively.

### 3.3. Stability Studies

Commercially available drug products having piracetam and levetiracetam were placed in stability chambers for 6-month duration in order to study stability profile of these drugs. ICH guideline was followed to comprehend both accelerated and long-term stability studies [28]. Stability study outcome suggested that all the drugs were stable in the described conditions with recoveries of almost 100% (Table 6).

### 3.4. Application of the Method to Pharmacokinetics

**Figure 9:**
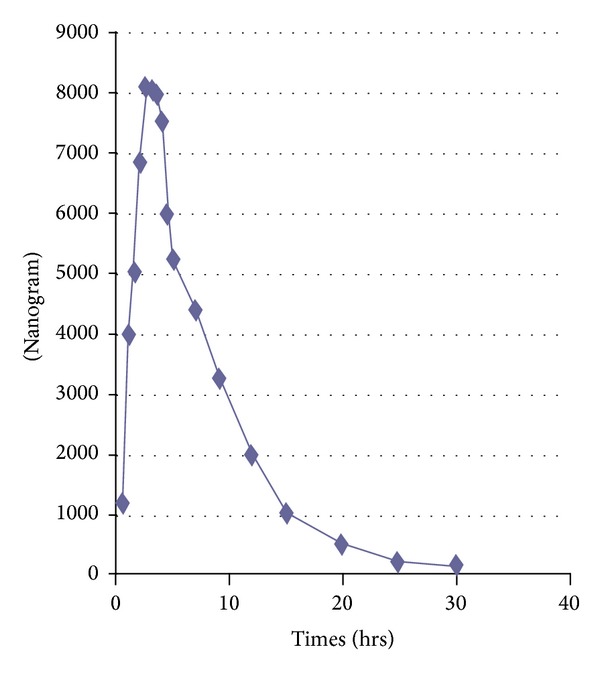
Mean plasma concentration-time profile after oral administration of piracetam 800 mg.

**Figure 10:**
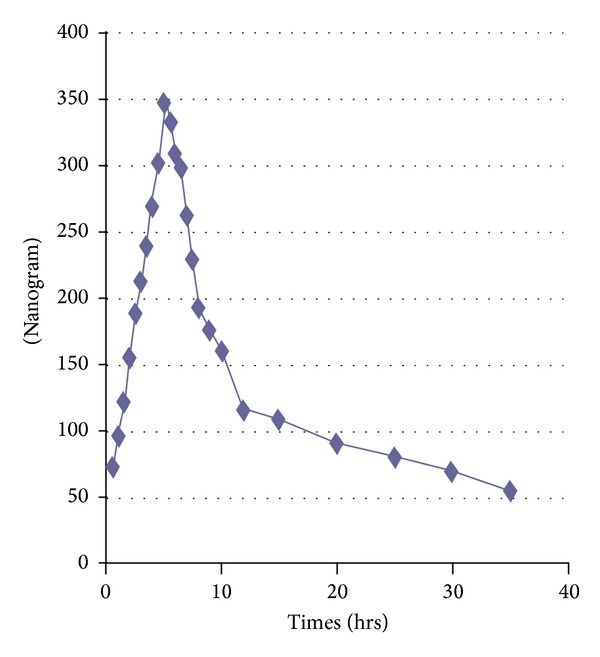
Mean plasma concentration-time profile after oral administration of levetiracetam 500 mg.

The proposed method was excellently suitable for pharmacokinetic study in human healthy Pakistani volunteers.  Figure 9 represents the mean plasma concentration-time curve after administration of a single 800 mg oral dose of piracetam while Figure 10 represents it for levetiracetam 500 mg single dose.

Results obtained for both the drugs piracetam and levetiracetam are comparable to the already reported pharmacokinetics study [29, 30] in human subjects.

## 4. Conclusion

The isocratic RP-HPLC method produced exhibited excellent resolution for piracetam and levetiracetam from its pharmaceuticals product, common excipients, and urine and serum samples. The method showed outstanding selectivity, precision, linearity, and sensitivity. The method has the stability indication power and can be used for the assay as well as for related substances determination of piracetam and levetiracetam.

## Figures and Tables

**Table 1:** Linearity of proposed method.

(ng/mL)	Piracetam		Levetiracetam	
Injected conc.	Recovered	%Recovery	Recovered	%Recovery
20	19.7	98.5	19.82	99.1
50	49.5	99	50.66	101.32
100	100.22	100.22	99.36	99.36
1000	999.1	99.91	998.47	99.847
5000	4978.9	99.578	4912.5	98.25
10000	9997.6	99.976	9993.41	99.9341

**Table 2:** Linear regression characteristic of proposed method.

	Piracetam	Levetiracetam
Linearity range (ng/mL)	20–10000	20–10000
Correlation coefficient	0.999	0.999
Slope	6864.07	6993.23
Intercept	−4322.01	−2311.11
LOQ	9.3	8.7
LOD	2.43	1.66

**Table 3:** Precision and accuracy of proposed method.

Injec. Con.	Recv. Con.	%Rec.	%RSD	Recv. Con.	%Rec.	%RSD
800	807	100.875	1.22	814	101.75	1.08
1000	1012	101.2	0.97	99.3	99.93	1.12
1200	1209	100.75	1.37	1193.7	99.475	1.45

**Table 4:** Repeatability and Reproducibility of Proposed method.

Conc. ng/mL	Intra-Day (%RSD)		Inter-Day (%RSD)	
	Piracetam	Levetiracetam	Piracetam	Levetiracetam
20	1.16	1.57	2.33	1.98
50	1.09	1.35	2.04	1.76
100	0.77	1.16	1.76	1.29
1000	1.24	1.28	1.29	1.34
5000	1.15	0.86	1.94	1.52
10000	0.96	1.08	1.24	1.07

**Table 5:** Recovery from serum and urine samples.

Conc. (ng/mL)	Serum		Urine	
	Piracetam	Levetiracetam	Piracetam	Levetiracetam
	%Recovery	%Recovery	%Recovery	%Recovery
20	97.6	96.8	98.74	97.59
50	97.9	97.4	99.26	98.37
100	99.2	98.8	98.05	98.14
1000	99.3	98.6	100.14	99.18
5000	97.4	96.9	99.93	97.33
10000	99.74	99.84	99.77	99.27

**Table 6:** Long term and real time stability studies by proposed method.

Test (claimed content)	Interval								
	Initial	1st month	2nd month	3rd month	4th month	5th month	6th month	Mean result	%RSD
Studies at accelerated condition (40°C + 75% H)									
PC (10 mg/5 mL)	99.68	100.76	99.96	99.45	98.26	98.43	98.25	99.255	0.979
LTC (10 mg/5 mL)	100.27	100.44	98.48	98.37	97.87	97.81	97.55	98.684	1.203

Stability studies at long term (30°C + 65% H)									
PC (10 mg/5 mL)	99.68	100.55	100.85	100.94	99.83	99.79	99.47	100.15	0.603
LTC (10 mg/5 mL)	100.27	100.62	99.78	98.38	98.18	98.04	97.96	99.032	1.159
